# Can we accurately predict where we look at paintings?

**DOI:** 10.1371/journal.pone.0239980

**Published:** 2020-10-09

**Authors:** Olivier Le Meur, Tugdual Le Pen, Rémi Cozot

**Affiliations:** 1 Univ Rennes, CNRS, IRISA, Rennes, France; 2 Univ Côte d’Opale, Calais, France; University of Pécs Medical School, HUNGARY

## Abstract

The objective of this study is to investigate and to simulate the gaze deployment of observers on paintings. For that purpose, we built a large eye tracking dataset composed of 150 paintings belonging to 5 art movements. We observed that the gaze deployment over the proposed paintings was very similar to the gaze deployment over natural scenes. Therefore, we evaluate existing saliency models and propose a new one which significantly outperforms the most recent deep-based saliency models. Thanks to this new saliency model, we can predict very accurately what are the salient areas of a painting. This opens new avenues for many image-based applications such as animation of paintings or transformation of a still painting into a video clip.

## Introduction

In the human brain, the processing of visual information requires up to 30% percent of the cortex, which is by far the most important when compared with other senses, such as touch and hearing. However, we are not able to process, at once, all visual information within our visual field. To deal with our limited visual processing resources, we have developed an active and highly dynamic process allowing us to sample our visual field. This process is called the visual attention [1].

Visual attention is composed of two different kinds of attention, namely overt and covert attention. The former is extremely interesting in the context of this study since this form of attention involves eye-movements. Therefore, the overt attention can be easily monitored thanks to the use of eye-tracking devices. The later form of attention, namely the covert attention, is more subtle since it does not involve eye-movements. The covert attention requires a volitional effort to direct our attention to a specific area of the visual field. This is clearly the case when we glance at something out of the corner of our eyes. This manifestation is not easily observable and would require to use event-related potentials or electroencephalography [2]. In this paper, we are then interested in the overt attention. It is also important to distinguish between bottom-up and top-down influences which account for our gaze deployment. The bottom-up attention is unconscious and does not require any conscious effort to move our gaze. This means that our gaze is effortlessly drawn by some parts of our visual field, which are salient. The definition of top-down is more tricky. Indeed, we can first consider that the top-down influences are related to the task at hand, as perfectly illustrated by the seminal study of Yarbus [3]. Depending on the task observers have to perform, the gaze deployment is significantly altered. Beyond the task at hand, top-down influences are also related to observers’ experience as well as their own characteristics such as age [4, 5] and their cultural experiences [6].

The computational modelling of visual attention mainly consists in determining in an automatic manner where an observer looks at [7]. This aims to simulate the overt bottom-up visual attention and therefore to explain the contributions of the visual features to the gaze deployment [8–10] Since the first models of visual attention [11, 12], a number of progress has been made. The performances of such models have significantly increased. This comes with the definition of new eye-tracking experiments allowing us to collect large scale eye-tracking dataset. New and efficient similarity metrics have been also defined to compare actual eye-tracking data with predicted one [13–15] More recently, a new generation of models, relying on deep networks, has brought a new momentum in this field of research, boosting our ability to predict salient areas [16–18]. Most of deep saliency models are trained with eye-tracking data collected over natural scenes. Such models perform best over this kind of visual scenes whereas their performances are significantly reduced when the input stimulus does not belong to such a category, such as webpages, UAV (Unmanned Aerial Vehicles) imagery [19], comics [20] to name a few. To cope with the lack of generalisation of visual attention models, it is common to fine-tune deep saliency models with eye-tracking data collected over the target visual scenes, such as comics [20] or webpages [21].

In this paper, we are interested in the design of a new deep saliency model to predict the overt bottom-up visual attention over paintings. For that purpose, we built a new eye-tracking dataset composed of 150 paintings stemming from five art periods, going from romanticism to fauvism periods. We first analyze the main characteristics of the visual deployment of observers while they freely viewed these paintings.

During the last decade, some studies investigated how the visual salience of paintings influences our gaze deployment. In [22], the authors investigated the influence of visual salience on abstract and depictive paintings. Two experiments were conducted, one in free-viewing and the other in target-search. The salience was estimated thanks to low-level visual features, such as color, luminance and orientation. The authors demonstrated that the low-level visual salience has a significant effect in attracting observers’ gaze in all conditions. In 2012, Massaro and his colleagues [23] went further by investigating both the influences of bottom-up and top-down processes on visual behavior. They observed that top-down processes prevailed over low-level visual bottom-up processes when paintings illustrate a human subject. Koide et al. [24] compared the visual deployment of novice and expert in art, while viewing paintings. They found significant differences between both populations. More specifically, fixations of experts are less driven by low-level features than those of novices, indicating that the visual deployment of experts in art relies more on high-level features than novice observers. In 2017, the authors [25] studied eye movements of children and adults looking at five Van Gogh paintings. As in the previous studies, authors tried to disentangle the bottom-up influences from the top-down ones. As expected, they found differences between children and adults [4, 5, 26]. Their results suggest that the bottom-up processes did not play a major role when adults viewed the paintings. The top-down processing is more important for adults than for children.

As for the aforementioned studies, we also investigate the ability of existing saliency models to predict where an observer look at. We expect that deep learning saliency models significantly outperform traditional (i.e. non-supervised and non-deep) saliency models, even if they have been trained on natural scenes. However, because of the poor generalization of existing saliency models when exposed to new kinds of stimuli, we believe we can go further. We then fine-tune an existing deep model to test whether or not we can improve its ability to predict where we look at.

The paper is organized as follows. First, we present the proposed eye tracking experiments conducted on 150 paintings. The second part presents the main gaze-based characteristics. We discuss whether or not they are similar to gaze-based characteristics computed on natural scenes. The third part evaluates the ability of computational models of visual attention to predict where we look at. We put to the test existing saliency models that are based either on handcrafted features or on deep networks. We also fine-tune a deep learning-based saliency model and we demonstrate an increase of the performance. We conclude the paper in the last section.

To sum up, our contributions are:

- the design of a new eye-tracking dataset over 150 paintings, belonging to five art periods;
- the analysis of gaze deployment over the proposed set of paintings;
- a benchmark of existing saliency models;
- a new and dedicated deep model for predicting saliency over paintings.

## Eye tracking experiment

In this section, we present the details of our eye tracking experiment.

### Stimuli

In painting history, there are many periods and movements. The 18^th^ century and early 19^th^ century are usually seen as a crucial period in which artists move from figurative realism to new ways for depicting the daily life. Indeed, during this short period, paint tubes made possible to directly paint *en plein air*, i.e. painting outside. Painting *en plein air* significantly changed the painting conditions [27] (e.g. limited set of materials, amount of details in the scene, changing environment, changing light, etc). The Romanticism [28] and Realism movements [29] emerged. The famous artists of Barbizon school are major actors of this period. Soon thereafter, photography appeared and caused concerns about painting and realism. If a painter skill is limited to copy details of a scene, photography tended to overcome this limitation. The ability to paint outside (*en plein air*) and the emergence of photography encouraged painters to go beyond photographic reality. Thereby Impressionism movement [30] focused more on visual feeling, while Pointillism [31] tried to produce more vibrant color. Finally, Fauvism movement [32] explored a non-naturalistic use of color. Nevertheless, these movements still belong to figurative painting in which the subject is still recognizable.

**Fig 1:**
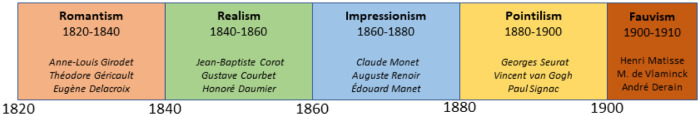
Main painting movements of 18*^th^* and early 19*^th^* century. The duration of each movement is approximately given. For each movement, we also give the name of some famous painters.

In this paper, we choose five art movements, namely realism, impressionism, pointillism, and fauvism. In addition to this, we also selected paintings from the romanticism period, not only for historical reasons but due to the willingness of romanticism painters to sublimate the beauty of nature in a realistic manner. Fig 1 presents a chronological view of the chosen art periods as well as famous painters for each of these periods.

The proposed dataset is composed of 150 paintings. Each of the 5 categories consists of 30 paintings. The titles of paintings used in this study are given in S1 File.

**Fig 2:**
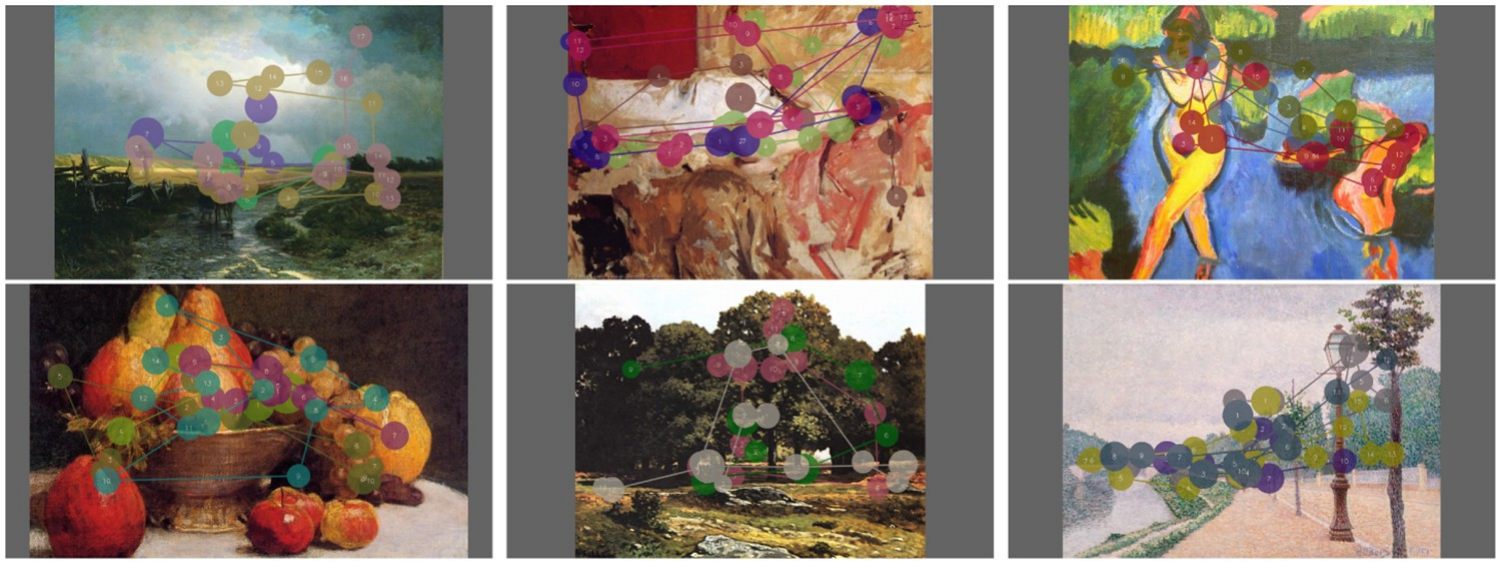
Examples of 4 scanpaths overlaid on paintings. The circles indicate the visual fixations. The number is the visual fixation index. From left to right: Vasilyev, *After a rain country road*, 1869; Sorolla, *Bacchante*, 1886; Pechstein, *Bank of a lake*, 1910; Fantin-Latour, *Bowl of fruits*, 1857; Sisley, *Chestnut avenue in la celle Saint Cloud*, 1865; Dubois-Pillet, *The Banks of the Seine at Neuilly, 1886*.

During the experiments, it was required to show paintings in a similar way. For that purpose, we used a grey image with a 16/9 ratio in which the painting is centered without any deformations. Left and right grey stripes are more or less important according the aspect ratio of paintings. Several examples are given in Fig 2. In addition, all paintings are in a landscape format.

We do not normalize the stimuli in luminance and contrast. The rationale of this choice is to be as close as the original paintings downloaded on Internet. However, for the sake of completeness, we report below the statistics concerning the Michelson contrast and average luminance and chrominance. We observe a significant difference in the average luminance for the five art movements, one-way ANOVA *F*(4, 140) = 8.00, *p* ≪0.05. Post hoc comparisons using the Tukey HSD test indicated that the average luminance for Impressionism period (*M* = 0.44, *SD* = 0.08) was significantly different from the average luminance for Romanticism period (*M* = 0.36, *SD* = 0.11). This is also the case between Realism and Pointillism, between Romanticism (*M* = 0.36, *SD* = 0.11) and Fauvism (*M* = 0.45, *SD* = 0.06), and between Romanticism (*M* = 0.36, *SD* = 0.11) and Pointillism (*M* = 0.48, *SD* = 0.07). Regarding the average chrominance (i.e. Blue and Red), we do not observe a significant difference between art movements, one-way ANOVA *F*(4, 140) = 0.28, *p* = 0.88, *F*(4, 140) = 1.04, *p* = 0.38, respectively. Regarding the contrast in luminance, we do not observe a significant difference between art movements, one-way ANOVA *F*(4, 140) = 1.21, *p* = 0.30.

### Apparatus and procedure

To perform the eye-tracking experiment, observers sit down in front of the screen. After a 9-point calibration session, paintings are displayed onscreen in a random manner both between subjects and stimuli. Stimuli are displayed for 4 seconds. Before each stimulus, a grey background is displayed for 2 seconds in-between. Any marker was used prior the onset of the stimulus in order both to guarantee a variety of starting points among obervers and to reduce the central bias [33] In order to limit the visual fatigue, the experiment is decomposed into 6 sessions during which 25 paintings are shown. Each session is preceded by a calibration phase.

A fixed-head SMI RED eye-tracker with a sampling frequency of 60Hz was used. Although this sampling frequency is low, it does not hinder the fixation-based analysis we aim to carry out in this study. However, it prevents us to make a saccade-based analysis. We recorded the guiding eye. The viewing distance was 87 cm and the diagonal of the screen was 56 cm. The screen subtended about 32° horizontally and 16° vertically. The screen resolution was 1600 × 800. The stimuli which were displayed in full-screen mode have a 1600 × 900 resolution. The number of pixel per degree of visual angle is then 49. A chin-rest was used in order to avoid any head movements and to increase the overall accuracy of collected data.

### Participants

Twenty one participants, 16 men and 5 women, took part in the experiments. Except one participant aged 50, all participants were aged between 20 and 30 year old. Participants were asked to look at paintings in a free-viewing task. The instruction given to participants was then to look at the paintings as naturally as possible.

In total, we collected in average 2100 fixations per participant, and overall more than 44000 fixations were collected.

The experiment has been conducted according to the principles expressed in the Declaration of Helsinki. Participants were properly instructed of the experiment goal and gave a verbal consent to participate in the experiment. Participant’s names were never recorded and eye tracking data were fully analyzed anonymously. For all these reasons, the approval of ethic committee was not required.

### Human saliency map

A common practice to infer human saliency map from eye tracking data is to compute first a fixation map. This map represents the collected fixations located on the definition space of the image, called in the following *Ω*. More formally, the fixation map $f:\Omega \subset R^{2}\to R^{+}$, where Ω = [1…*N*] × [1…*M*] with *N* and *M* the resolution of the input stimulus [34], is defined as below:

$$\begin{array}{c} f\left(x\right)=\sum_{i=1}^{K}\delta \left(x-x_{i}\right)\times \tau \left(x_{i}\right) \end{array}$$ (1)

where, **x**_*i*_ represents the 2D spatial position of the *i*^*th*^ fixation, *K* is the total number of fixations, *δ* is the Kronecker delta, such that *δ*(*a*) is 1 if *a* = 0, 0 otherwise. *τ*(**x**_*i*_) is a positive weight applied to the current fixated location. In the classical approach, we consider that all fixations have the same weight, i.e. *τ*(**x**_*i*_) = 1, ∀*i*.

The fixation map is then convolved with a 2D isotropic Gaussian function *G*_*σ*_ [13, 34] to produce a continuous saliency map *S* ($S:\Omega \subset R^{2}\to \left[0,1\right]$ (or [0, 255] for the sake of the visualization)):

$$\begin{array}{c} S\left(x\right)=N(f\left(x\right)*G_{\sigma }\left(x\right)) \end{array}$$ (2)

where, N is a peak-to-peak normalization operator. *G*_*σ*_ is an isotropic 2D Gaussian kernel. The standard deviation *σ*, expressed in pixel, shall represent the number of pixels falling into the fovea; in this case, *σ* represents one degree of visual angle, i.e. 49 pixels.

## Results and analysis

In this section, we present the analysis of the eye tracking data we collected.

### Scanpath and heat map visualization

Fig 2 illustrates four scanpaths overlaid on six paintings. The scanpaths are composed of fixations, illustrated thanks to circles, and saccades, represented by the straight line joining two fixation points. In the following, we analyze the distribution of fixation durations as well as saccade amplitudes.

**Fig 3:**
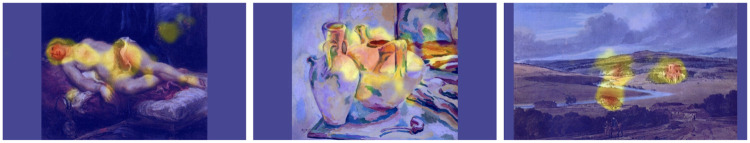
Examples of heat maps for 3 paintings. From left to right: Delacroix, *Odalisque*, 1825; Georges Braque, *Still life with jugs and pipe*, 1906; Girtin Kirkstall, *Abbey Yorkshire*, 1801.

Fig 3 illustrates some heat maps. These maps are color representation of saliency maps. They are very convenient to quickly determine where observers look at. The reddish parts correspond to the most visually salient areas.

### Gaze-based characteristics

**Fig 4:**
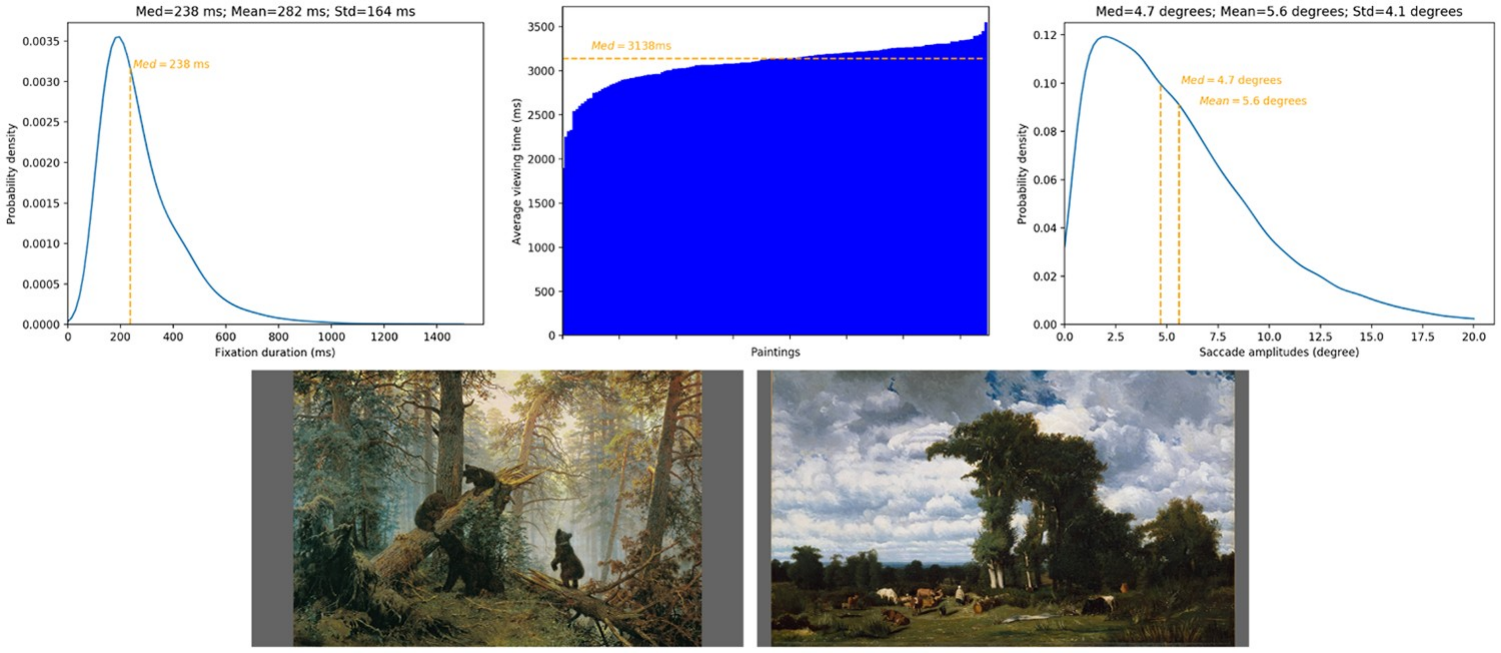
(Top) Fixation durations (left), average fixation time per paintings, sorted in ascending order (middlle) and the distribution of saccade amplitudes (right). (Bottom) Highest fixation time for *Morning in a pine forest*, Ivan Shishkin, 1889 (left) and shortest fixation time for *Paysage avec du betail au limousin, Jules Dupre, 1837. (right)*

Fig 4 illustrates the distribution of fixation durations (on the left), the average fixation time per painting (on the middle) and the distribution of saccade amplitudes (on the right).

We observe that the distribution of visual fixation durations follows a long-tailed asymmetric distribution. The median fixation duration is equal to 238 ms. These observations are similar to what researchers are used to observe on natural scenes [35]. We also examine the total fixation time, which is the sum of fixation durations over the paintings divided by the number of observers. On Fig 4, we sort in ascending order the fixation time. The painting which has the highest fixation time, equal to 3550 ms, is *Morning in a pine forest*, Ivan Shishkin, 1889. The painting with the shortest fixation time, equal to 1896 ms, is *Paysage avec du betail au limousin*, Jules Dupre, 1837. These paintings are illustrated on the bottom of Fig 4 One obvious difference between these 2 paintings concerns the number and the size of the visually important areas. In the former, there are 4 regions of interest, i.e. the four bears. They are in close proximity to each other and located on the bottom center of the paintings. Regarding the latter painting, the number of visually important areas is much higher than for the previous painting. In addition, except the two central big trees, these visually important areas are small and occupied a large space horizontally. These previous observations could explain why the viewing time is so small for the paintings *Paysage avec du betail au limousin*, Jules Dupre, 1837. In order to get the maximum information during the 4 seconds of viewing, observers may jump quickly from one area to another. This strategy would reduce the fixation duration and would allow observers to scan the whole painting. A one-way ANOVA was conducted to compare the effect of art movements on fixation time. Result indicates that there is no significant effect of art movements on the fixation time *F*(4, 140) = 0.69, *p* = 0.59.

The distribution of saccade amplitudes is a long-tailed asymmetric distribution, as classically reported in the literature, which could be easily simulated by a Gamma distribution [36]. The median saccade amplitude is equal to 4.6 degrees of visual angle.

**Fig 5:**
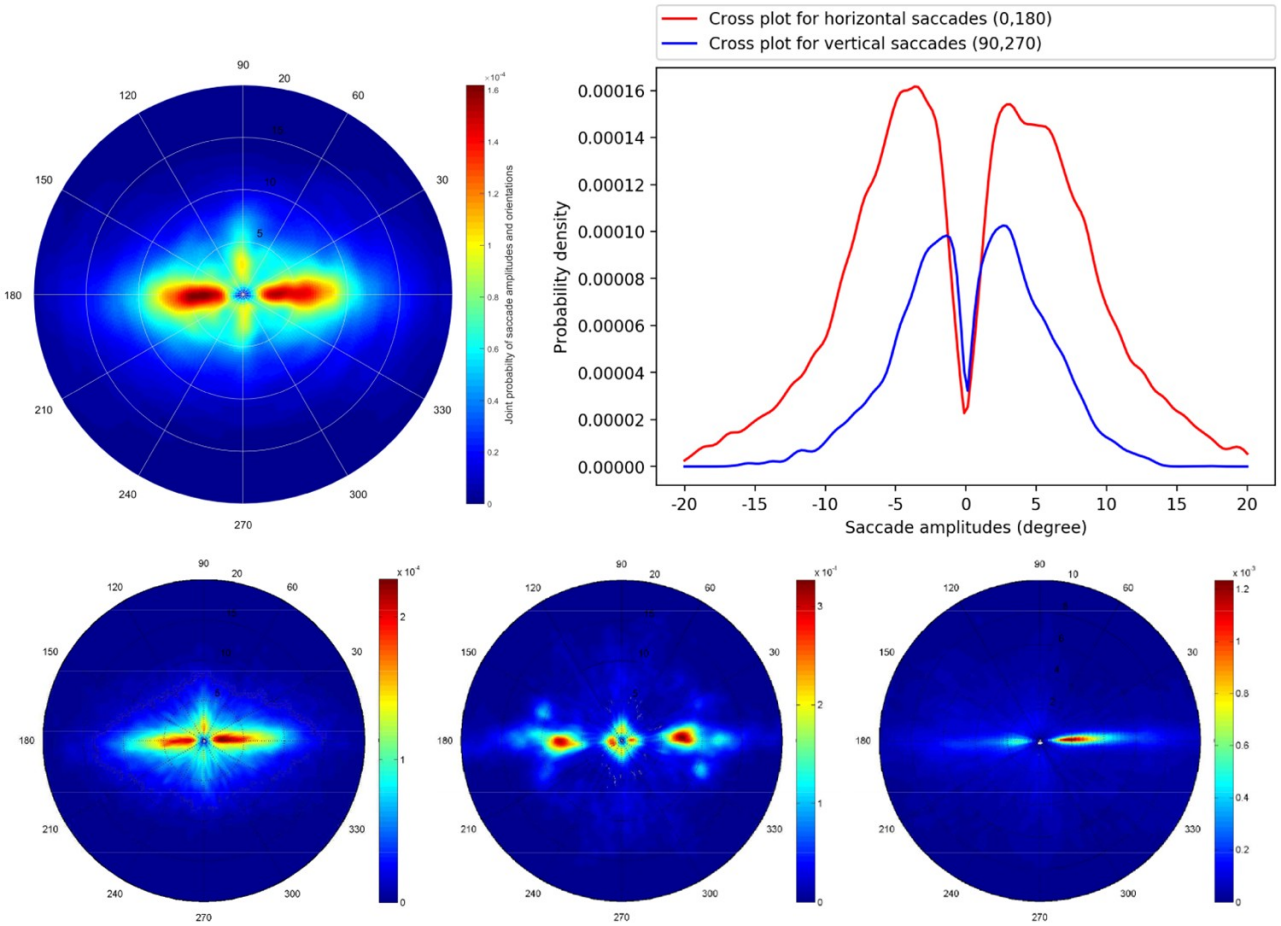
Joint distribution of saccade amplitudes and orientations (Top-left). Horizontal and vertical cross sections of the probability distribution for horizontal saccades (red plot) and vertical saccades (blue plot) in function of the saccade amplitudes, respectively (top-right). On the bottom, the joint distributions for natural scenes, conversational videos and webpages are illustrated (adapted from [37, 38]).

Fig 5 presents the polar plot of the joint distribution of saccade amplitudes and orientations. The radial axis gives the saccade amplitude in degrees whereas the angular coordinate represents the saccade orientation. We observe a strong horizontal bias, indicating that observers preferably moves their eyes along the horizontal axis. There are much more horizontal saccades than vertical ones. We compare the observed joint distribution with distributions computed over natural scenes, conversational videos and webpages as proposed in [37, 38]. These distributions are illustrated on the bottom of Fig 5. Qualitatively speaking, the joint distribution of saccade amplitudes and orientations observed on paintings is close to the distribution computed over natural scenes. To objectively assess the similarity between distributions, we compute the correlation coefficient as well as the Kullback-Leibler divergence between the paintings joint distribution and distributions computed over natural scenes, conversational videos and webpages. The correlation coefficients are all positive and highly significant (*p* ≪0.05); they are equal to 0.93, 0.67 and 0.62, respectively. The Kullback-Leibler scores are equal to 0.07, 0.31 and 0.29, respectively. These scores support the observation that the gaze deployment over the proposed paintings is very similar to the gaze deployment over natural scenes.

**Fig 6:**
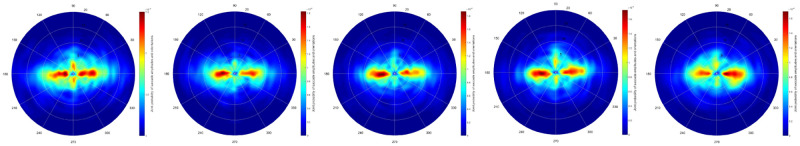
Joint distribution of saccade amplitudes and orientations for the five periods, e.g. Romanticism, Realism, Impressionism, Pointillism and Fauvism, are illustrated.

Fig 6 illustrates the joint distributions of saccade amplitudes and orientations for the five art movements independently. We observe a strong horizontal bias for the five art movements. There was a positive correlation between the different art movements (see Table 1); all correlation values are highly significant, *p* ≪ 0.05.

**Table 1:** Correlation coefficient between joint distributions of saccade amplitudes and orientations.

	Romanticism	Realism	Impressionism	Pointillism	Fauvism
Romanticism	1.00	0.96	0.95	0.96	0.94
Realism	0.96	1.00	0.97	0.95	0.94
Impressionism	0.95	0.97	1.00	0.96	0.95
Pointillism	0.96	0.95	0.96	1.00	0.94
Fauvism	0.94	0.94	0.95	0.94	1.00

Table 2 presents fixation durations and saccade amplitudes per art movement. The average fixation duration for the 5 paintings categories is equal to 285, 286, 283 and 279 ms for Romanticism, Realism, Impressionism, Pointillism and Fauvism, respectively. A one-way ANOVA was conducted to compare the effect of art movements on fixation durations. There was no significant effect of art movements on fixation durations for the five art movements *F*(4, 33230) = 1.98, *p* = 0.09.

**Table 2:** Fixation durations and saccade amplitudes per art movement. The average, standard deviation and number of fixations/saccades are reported.

	Romanticism	Realism	Impressionism	Pointillism	Fauvism
Fixation					
Duration	285±164	286±161	281±166	283±166	279±165
Number	6676	6698	6496	6713	6652
Saccade					
Amplitude	5.27±4.12	5.14±3.94	5.41±4.09	5.19±3.85	5.25±3.73

Concerning the average saccade amplitudes, they vary between 5.1 and 5.4 degrees of visual angle, as indicated in Table 2. A one-way ANOVA was conducted to compare the effect of art movements on saccade amplitudes. There was a significant effect of art movements on saccade amplitudes for the five art movements *F*(4, 30188) = 4.05, *p* = 0.002. Post hoc comparisons using the Tukey HSD test indicated that the mean saccade amplitudes for Realism period (*M* = 5.14, *SD* = 3.94) was significantly different than the saccade amplitudes for Impressionism period (*M* = 5.41, *SD* = 4.09). A significant difference is also observed between saccade amplitudes of Impressionism period (*M* = 5.41, *SD* = 4.09) and saccade amplitudes of Pointillism period (*M* = 5.19, *SD* = 3.85).

### Saliency distribution in paintings

**Fig 7:**
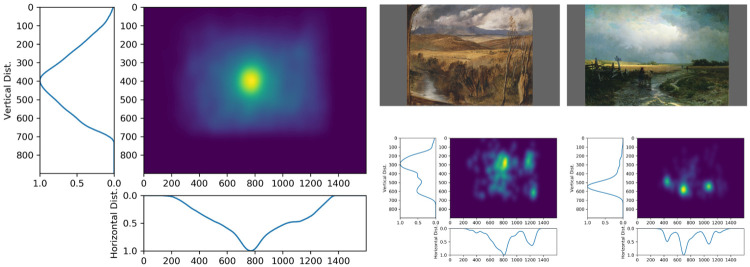
Saliency distribution on paintings. On the left, the average saliency computed over all paintings. On the right, two examples of saliency distribution for the paintings (Landseer, *A highland landscape*, 1830; Vasilyev, *After a rain country road*, 1869).

Fig 7 presents the average saliency distribution of salience (on the left) and two examples on two paintings (on the right).

When we aggregate all human saliency maps, we observe that there is a strong center bias. This observation was common on natural scenes, for which observers tend to look towards the screen center, whatever the salience [33, 39]. For paintings, a similar trend is observed. The marginal vertical and horizontal saliency distributions, on the bottom and the left-hand side respectively, present a strong peak near the center of the image. This observation is not so surprising since the painting category and the scene layout are rather similar to what we observe on natural scenes.

### Inter-Observers Congruency (IOC)

In this section, we evaluate the congruency between obervers. The IOC score reflects the congruence or the variability among different observers looking at the same stimulus. We follow the procedure described in [40] and in [41].

The computation process of IOC consists of several steps. First the saliency map of all observers except one is computed in a leave-one-out fashion. This saliency map is then binary thresholded to keep the top 25% most salient pixels. Then the percentage of the excluded observer’s fixations that fall into the thresholded salient areas is determined. For a given stimulus, the final IOC score is the harmonic mean of the scores of all observers. This score is in the range [0, 1], where 0 indicates the lowest congruency (or the highest dispersion) and 1 indicates the highest congruence (or the lowest dispersion). In the latter case, it would mean that all observers have exactly looked at the same areas, but not necessarily in the same order.

**Fig 8:**
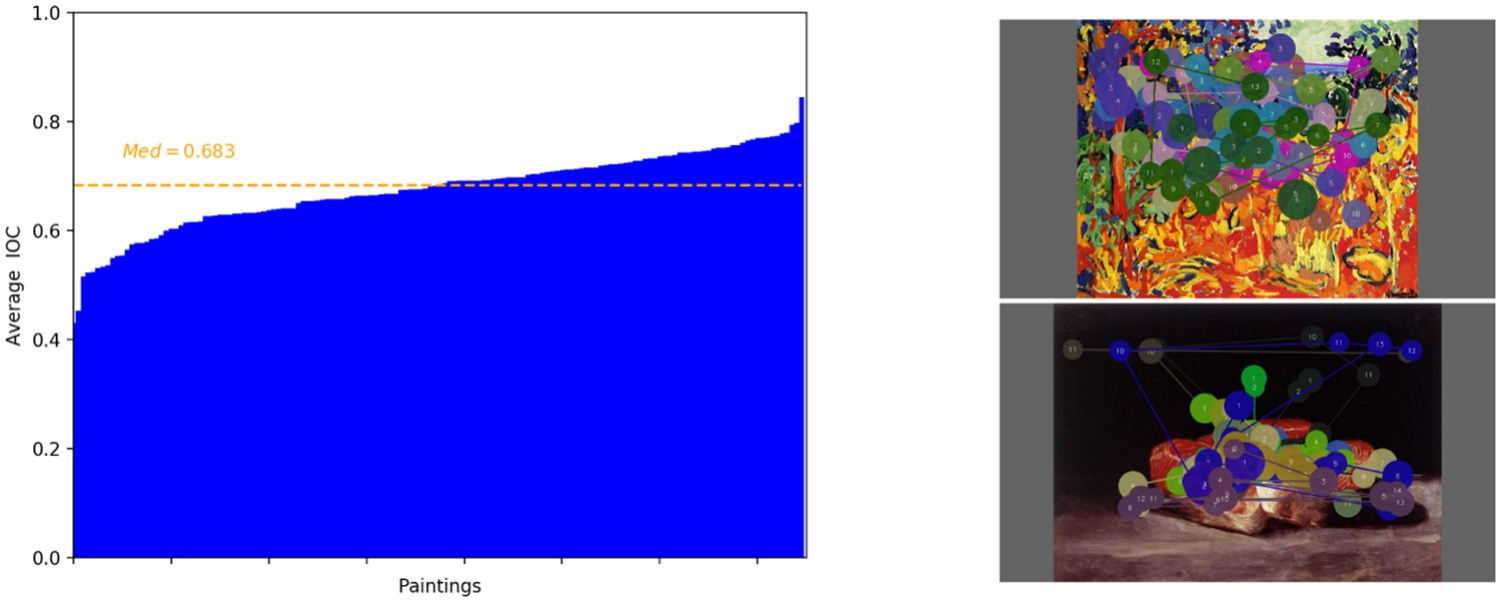
On the left: Inter-Observers Congruency (IOC) per paintings. On the right: the painting (*The Orchard*, Vlaminck, 1905) having the lowest (top) and the highest IOC (*Bodegon con salmon*, Goya, 1812) (bottom).

Fig 8 gives the average IOC per painting, sorted in ascending order. The median value is 0.683. The lowest is equal to 0.433, for the painting *The Orchard*, Vlaminck, 1905 (Fauvism period). The highest value is 0.844, for the painting *Bodegon con salmon*, Goya, 1812 (Romanticism period). Scanpaths for these two paintings are illustrated on the right hand side of Fig 8. As expected, the lowest agreement between observers is observed for a painting containing a number of visual information, very rich, colorful and quite complex to analyze in a glance (Fig 8, top (right-hand side)). This painting invites observers to explore and to find out details. At the opposite the painting having the highest IOC is rather simple and contains an unique object standing from the background. Observers, except one who looked in the background, focused on the object in the foreground (Fig 8, top (right-hand side)).

We also perform the IOC analysis per art movement. The average IOC scores and their standard deviations are 0.67±0.07, 0.68±0.05, 0.61±0.14, 0.58±0.14 and 0.62±0.14, for Romanticism, Realism, Impressionism, Pointillism and Fauvism, respectively. A one-way ANOVA was therefore conducted to compare the effect of art movements on the IOC scores. There was a significant effect of art movements on the inter-observers congruency for the five art movements *F*(4, 140) = 3.47, *p* = 0.009. Post hoc comparisons using the Tukey HSD test (*p* < 0.05) indicated that the IOC scores for Romanticism period (*M* = 0.67, *SD* = 0.07) was significantly different than the IOC scores for Pointillism period (*M* = 0.58, *SD* = 0.14). A similar observation is made between Realism (*M* = 0.68, *SD* = 0.05) and Pointillism periods (*M* = 0.58, *SD* = 0.14). In addition, the lowest average IOC score is observed for the Pointillism period. These results underline that the Pointillism painting style, which consists in placing small and distinct colors next to each other to form an image, affects the visual gaze deployment. It could be due to the visual complexity of this style, which could alter our ability to interpret the visual scene. For a good understanding of such paintings, more visual information might be required to get the whole meaning of the scene. However, deeper analysis would be required to draw a definitive conclusion regarding this observation. From these results, we can also assume that it would be more difficult to predict the salient areas on paintings belonging to the Pointillism period. In the next section, we verify this assumption by evaluating saliency models.

## Do computational models of visual attention predict well the salience of paintings?

In this section, we evaluate the ability of existing saliency models to predict where observers look at when they freely-view paintings displayed onscreen. We also tailor an existing model to predict the salience of paintings.

### Method

To carry out the evaluation, we use quality metrics used in the MIT benchmark [14]:

- Correlation Coefficient, *CC* ∈ [−1, 1], evaluates the degree of linearity between two saliency maps. *CC* = 1 indicates that there is a perfect linear relationship between the two maps;
- SIM, *SIM* ∈ [0, 1], represents the similarity between two saliency map distributions, evaluated through the intersection between histograms of saliency. *SIM* = 1 indicates the highest similarity;
- AUC, *AUC* ∈ [0, 1], is the area under the Receiver Operating Characteristics (ROC) curve. We classically use two implementations of AUC, namely AUC-J and AUC-B. Both metrics measure how well the predicted saliency map of an image predicts the ground truth human fixations on the image. The AUC is determined by plotting the ROC curve thanks to binary thresholdings. The difference between AUC-J and AUC-B relies on how true and false positives are calculated.
- KL, *KL* ∈ [0, + ∞[, is the Kullback Leibler divergence between the predicted and the human saliency maps. *KL* = 0 indicates a perfect similarity between the two maps.

More details about these metrics can be found in [13, 14, 42].

We evaluated 4 non-supervised handcrafted-based and 4 deep learning-based saliency models. The 4 non-supervised models are GBVS [43], RARE2012 [44], AIM [45] and AWS [46]. The 4 deep learning-based models are MLNET [47], deepGAZEII [16], SALICON [17], SAM-VGG and SAM-ResNet [18]. Table 3 presents the main characteristics of the four tested deep models. All of them rely on a deep network dedicated for object recognition, such as VGG-16/19 [48] and ResNet [49]. The main idea behind the proposed architectures is to leverage these CNN in order to extract deep features; these features are then used to determine the salient part of an image. For this purposed, different architectures have been proposed. They could be multiscale, such as SALICON, or involve a shallow network such as MLNET and DeepGazeII. SAM models leverage attentive Convolutional LSTM (Long Short-Term Memory) to enhance saliency features. Regarding the loss function, MLNET used a weighted Euclidean distance in order to give more importance to errors on salient areas. SAM models used a combination of saliency-based losses, which turns out to be very efficient [50]. The datasets used to train these models are all composed of natural scenes. Note that SALICON dataset does not consist of eye-tracking data but of mouse tracking data. Another interesting point to underline is the number of trainable parameters. As given by Table 3, SAM-ResNet model has the highest number of trainable parameters (≈ 70 Millions) whereas MLNET has the lowest number of trainable parameters (≈ 15 Millions).

**Table 3:** Main characteristics of the tested deep models.

Model	Year	Architecture	Training
SALICON	2015	Based on CNN for object recognition	MIT1003 eye dataset
		Two streams (coarse/fine)	OSIE eye dataset
		≈ 29 Mill. of parameters	NUSEF / FIFA
			PASCAL-S / Toronto
MLNET	2016	Based on VGG-16	SALICON dataset (Mouse)
		Shallow CNN	MIT300 eye dataset
		1280 VGG feature maps	Weighted loss function
		Learned prior	
		≈ 15 Mill. of parameters	
DeepGazeII	2016	Based on VGG-16	SALICON dataset (Mouse)
		Shallow CNN	MIT1003 eye dataset
		2560 VGG feature maps	
		Gaussian bias	
		≈ 20 Mill. of parameters	
SAM (VGG/ResNet)	2018	Based on VGG-16/ResNet50	SALICON dataset (Mouse)
		LSTM network	MIT300/1003
		Learned priors	CAT2000
		SAM-VGG, ≈ 50 Mill. of parameters	Saliency-based loss
		SAM-ResNet, ≈ 70 Mill. of parameters	

### Qualitative analysis

**Fig 9:**
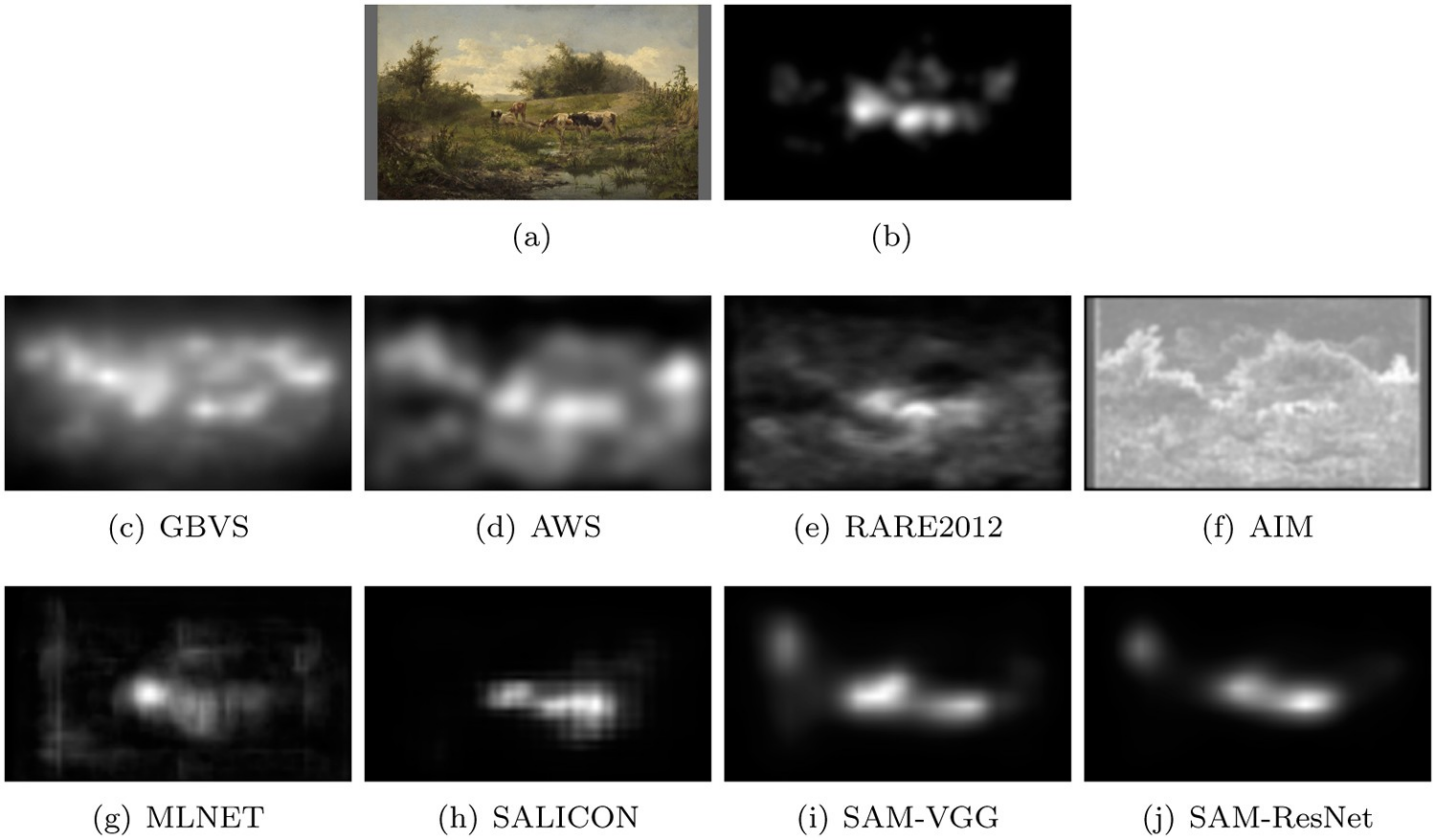
Saliency maps from non deep (second row) and deep models (third row). The first row illustrates the original stimulus (Bilders, *Cows at a pond, 1856)* and its human saliency map.

Fig 9 presents a subjective comparison between human and predicted saliency maps. On the first row, the original image and the human saliency map are shown. The second row presents saliency maps computed with the four non-supervised saliency models. The last row illustrates the saliency maps predicted by deep models.

It is noticeable that deep saliency maps are much more focussed than non deep saliency maps. They, in addition, seem much more similar to the human saliency map than non deep saliency maps. To make this point clear, we proceed in the next section to a quantitative analysis of the similarity degree between human and predicted saliency maps.

### Quantitative analysis

Table 4 presents the performances of the tested saliency models. Several conclusions can be drawn.

**Table 4:** Performances of saliency models on paintings dataset.

Model	CC ↑	KL ↓	SIM ↑	NSS ↑	AUC-B ↑	AUC-J ↑
GBVS	0.506	0.962	0.446	1.256	**0.809**	0.817
RARE2012	0.443	1.020	0.438	1.103	0.777	0.786
AIM	0.315	1.245	0.371	0.772	0.723	0.735
AWS	0.427	1.045	0.430	1.083	0.762	0.769
Mean	0.422	1.068	0.421	1.053	0.774	0.776
MLNET	0.576	**0.832**	0.513	1.524	0.770	0.818
DeepGazeII	0.485	0.896	0.488	1.394	0.679	0.804
SALICON	0.538	0.880	0.517	1.445	0.708	0.827
SAM-ResNet	**0.700**	0.984	**0.613**	**1.834**	0.782	**0.862**
SAM-VGG	0.617	0.970	0.561	1.603	0.752	0.846
Mean	0.583	0.912	0.551	1.560	0.738	0.831

We first observe that the performances of the 4 deep learning-based saliency models are, as expected, much better than the 4 non-supervised handcrafted-based models. The deep models perform on average at 0.583 in terms of correlation coefficient whereas the handcrafted models perform at 0.422. This observation holds true for all metrics except the AUC-B metrics. When dealing with natural scenes, the clear advantage of deep models over non-supervised has been reported in many studies, such as [51]. In this study, we observe similar conclusions but for paintings.

The best non deep model is GBVS whereas the best deep model is SAM-ResNet. The difference in *CC* scores, *CC* = 0.506 and *CC* = 0.700 for GBVS and SAM-ResNet respectively, is statistically significant (paired t-test, *t*(149) = −17.28, *p* ≪0.05).

Regarding more specifically deep models, the best model is clearly SAM-ResNet [18], for which the correlation coefficient is equal on average to 0.7. The best prediction gets a correlation of 0.905 whereas the worst prediction gets a correlation of 0.275. SAM-ResNet outperforms significantly MLNET (paired t-test, *t*(149) = 9.68, *p* ≪0.05), DeepGazeII (paired t-test, *t*(149) = 15.06, *p* ≪0.05), SALICON (paired t-test, *t*(149) = 10.73, *p* ≪0.05), SAM-VGG (paired t-test, *t*(149) = 10.23, *p* ≪0.05) models. The good performance of SAM-ResNet can be explained by its high number of trainable parameters, its learned priors and its loss function which leverages a combination of saliency metrics. All these points could provide to SAM-ResNet a better generalization than other tested models.

As mentioned earlier, deep saliency models perform quite well on average on the proposed paintings dataset. This is eventually not that surprising since the chosen paintings do not violate ecological visual principles. Those paintings aim at representing casual objects, natural scenes and characters with more or less visual fidelity. It suggests that deep models, that has been trained over natural scenes, are not impeded by neither the painting style nor the limitations imposed by painting materials [22]. The deep models then generalize well and significantly outperform non-supervised handcrafted-based models by successfully leveraging low-level features and semantics (or higher-level features) ones [52]. This is consistent with findings in [22], supporting the bottom-up hypothesis of salience-driven attention for the tested paintings.

However, this observation needs to be tone down. Indeed, performances of deep models are not that high compared to those we are used to observe on natural scenes. For instance, the model SAM-ResNet performs, in terms of *CC*, at 0.78 on MIT300 [14], and at 0.89 on CAT2000 dataset [53] (these scores have been taken from the MIT benchmark website https://saliency.mit.edu/). On the proposed dataset, the performance of this model decreases to 0.7. Similarly, MLNET performs at 0.67 on MIT300, whereas it performs at 0.576 on the proposed dataset. This suggest that there is room for improvement and that we can go further by improving the ability of such models to predict the salience over paintings.

To go deeper into the analysis, we also evaluate the performances for the five styles, namely Fauvism, Impressionism, Pointillism, Realism, and Romanticism. For this test, the previous five deep models are evaluated. Table 5 presents the results for CC, NSS and AUC-J.

**Table 5:** Performances of deep models on the 5 art periods.

Model	Style	CC ↑	NSS ↑	AUC-J ↑
MLNET	Fauvism	0.600	1.553	0.825
	Impressionism	0.533	1.367	0.798
	Pointillism	0.536	1.306	0.802
	Realism	**0.601**	**1.709**	**0.828**
	Romanticism	0.564	1.564	0.824
DeepGazeII	Fauvism	0.485	1.394	0.804
	Impressionism	0.460	1.305	0.796
	Pointillism	0.452	1.206	0.782
	Realism	0.492	1.609	0.823
	Romanticism	0.462	1.444	0.807
SALICON	Fauvism	0.498	1.271	0.820
	Impressionism	0.523	1.362	0.809
	Pointillism	0.497	1.234	0.809
	Realism	**0.577**	**1.693**	**0.842**
	Romanticism	0.576	1.572	0.842
SAM-ResNet	Fauvism	**0.723**	1.834	0.867
	Impressionism	0.648	1.659	0.845
	Pointillism	0.704	1.663	0.856
	Realism	0.701	**2.015**	0.862
	Romanticism	0.705	1.949	**0.874**
SAM-VGG	Fauvism	**0.647**	1.635	0.849
	Impressionism	0.571	1.446	0.831
	Pointillism	0.620	1.464	0.837
	Realism	0.623	**1.757**	0.848
	Romanticism	0.621	1.707	**0.857**

Overall, deep saliency models perform rather well on the 5 art movements. The highest correlation coefficient is 0.723 (SAM-ResNet for the Fauvism period) whereas the lowest is 0.460 (DeepGazeII for the Impressionism period). Still in terms of correlation coefficient, the best deep model, over the five periods, is SAM-ResNet. It performs well over Fauvism, Pointillism, Realism and Romanticism. The lowest performances are observed on Impressionism.

It is also interesting to emphasize that the performances of MLNET and SALICON, and to a lesser extent SAM-ResNet and SAM-VGG, are the highest for paintings of the Realism and Romanticism periods. Realism artistic movement aims to portray real and typical contemporary people and situations by taking care to be as close as possible to truth and accuracy. Such paintings depicted everyday subjects and situations in contemporary settings, and attempted to depict individuals of all social classes in a similar manner [54]. Romanticism period emphasized intense emotion as an authentic source of aesthetic experience, placing new emphasis on such emotions as apprehension, horror and terror, and awe [55]. The performance of deep-based models on these two art movements could be explained by the fact that deep models have been trained over natural scenes, depicting the daily life. For instance, MLNET model has been trained over SALICON dataset [56] and MIT300 dataset [57], whereas SAM-ResNet and SAM-VGG have been trained over 4 datasets, i.e. SALICON dataset [56], MIT1003 dataset [58], CAT2000 dataset [53] and MIT300 dataset [57], as given in Table 3.

The art movements for which deep models perform least are the Pointillism and Impressionism movements. This observation could be explained by the art history. Indeed, one key factor that usually explains the emergence of Impressionism, is the arrival of photography that questions artists about their own works. In a kind of opposition to photography *mechanical* realism, the impressionist painters do not try to copy reality; they rather try to create images that depict their own visual perception and feeling. Less importance is then given to realism and details whereas the focus is set on visual feeling. Pointillism, that belongs to neo-impressionism, also proposes an approach to differentiate painting from photography realism. Rather than focusing on impression, pointillism painters use small dots of pure colors to produce a more vibrant color than legacy painting and photography. This observation is however to tone down since we do not observe a significant influence of the art movement on the correlation coefficient for SAM-ResNet model (one-way ANOVA *F*(4, 140) = 1.45, *p* = 0.22).

### Can we go further?

To test whether or not we can improve the performance of prediction, we fine-tune the best performing model on the paintings dataset, namely SAM-ResNet. We have chosen SAM-ResNet for several reasons. First, this is the model that performs the best over the proposed paintings dataset as presented in the previous section. As it already performs rather well, the challenge to improve it is then more difficult. Second, we believe that SAM-ResNet architecture has some advantages compared to other deep models, such as the priors that are learned, and the loss function which leverages both saliency maps and fixation maps. Obviously, its high number of trainable parameters is also interesting to tailor the model to paintings. For fine-tuning SAM-ResNet, we split the paintings dataset into a training set, composed of 90 paintings randomly chosen, a validation set of 20 paintings, and a test set composed of 40 paintings.

Table 6 presents the performances of SAM-ResNet model after fine-tuning. Performances are evaluated over the test dataset. We then recompute SAM-ResNet performance on the test dataset (first line of Table 6). Results indicate that SAM-ResNet model fine-tuned with paintings dataset performs much better than the original version; for the correlation coefficient, the difference is significant, paired t-test, *t*(39) = −3.17, *p* ≪0.005.

**Table 6:** Performances of SAM-ResNet after fine-tuning on the test dataset.

Model	CC ↑	KL ↓	SIM ↑	NSS ↑	AUC-B ↑	AUC-J ↑
SAM-ResNet	0.69	1.08	0.60	1.79	0.78	0.85
SAM-ResNet fine-tuned	0.75	0.83	0.68	1.92	0.84	0.88
Min.	0.58	0.33	0.56	1.30	0.76	0.81
Max.	0.89	3.00	0.77	2.72	0.89	0.92
Gain (%)	+9.7%	-23%	+11.8%	+7.2%	+7.5%	+2.9%

**Fig 10:**
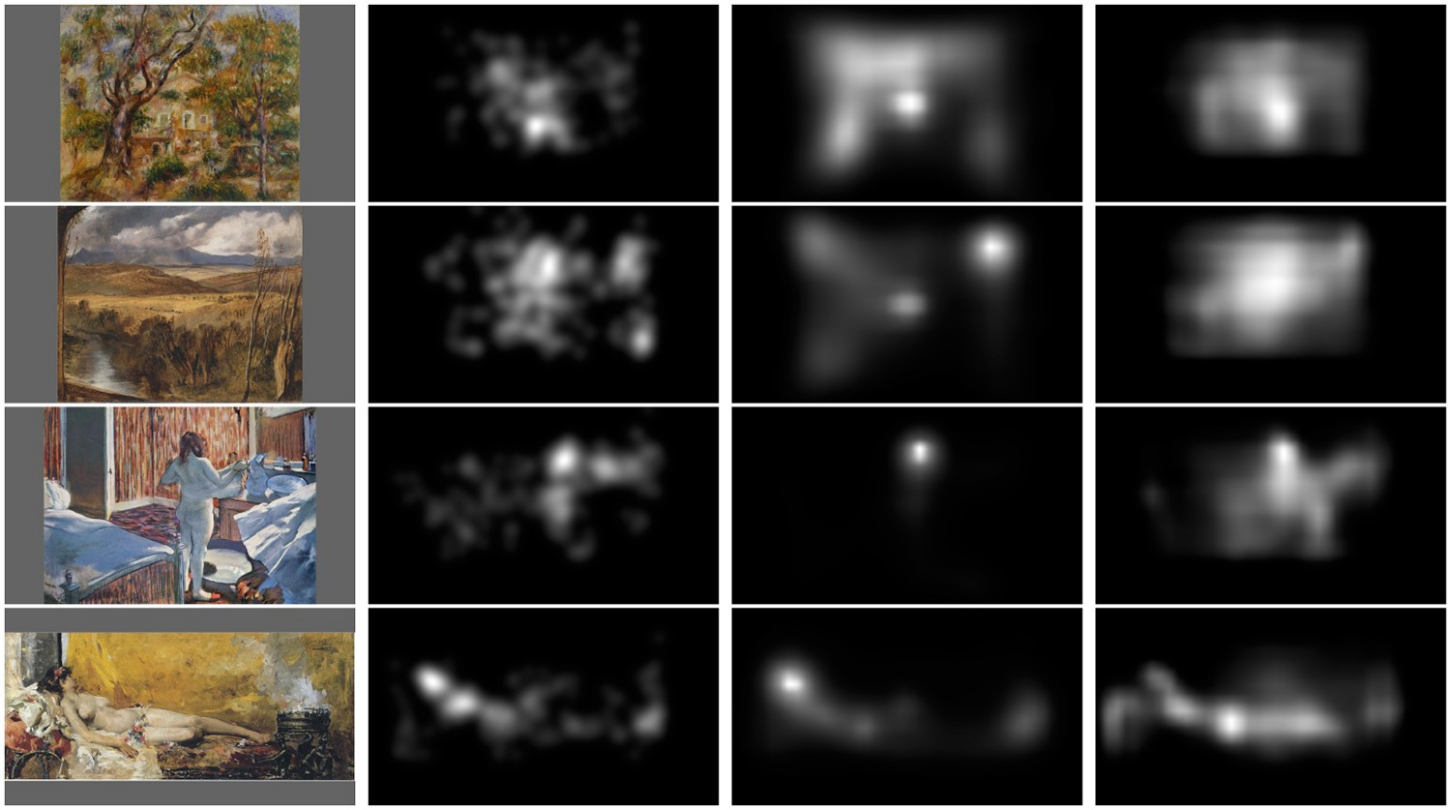
From left to right: original painting, human saliency map, SAM-ResNet prediction, and fine-tuned SAM-ResNet prediction. First row: Renoir, *La ferme des Collettes*, 1908. Second row: Landseer, *A highland landscape*, 1830. Third row: Degas, *Woman at her toilette*, 1877. Fourth row: Sorolla, *Resting bacchante*, 1887.

Fig 10 illustrates saliency maps predicted from the original and the fine-tuned deep model. We observe that the fine-tuned model provides less focused maps and tends to detect more salient areas compared to the original one. The fact that saliency maps are less focused allows to be closer to human maps. On the top, for Renoir paintings, the correlation increases from 0.574 to 0.807. For the Landseer paintings (second row), the gain in correlation is also significant; the *CC* score increases from 0.529 to 0.804. For Degas painting (third row), we also observe a significant increase of the *CC* score, from 0.54 to 0.749. Over the 40 tested paintings, the correlation increases for 28 paintings and decreases for 12 paintings. The average increase (resp. decrease) is equal to 0.135 and 0.09. The most important gain equal to 0.29 is observed for Sisley painting (*Chestnut avenue in la celle Saint-Cloud*, 1865). The most important regression is equal to 0.14 and observed for Sorolla painting, (*Resting Bacchante*, 1887). The coefficient of correlation decreases in this case from 0.759 to 0.612. The corresponding saliency maps are illustrated at the bottom of Fig 10. The original version of SAM-ResNet succeeds in better detecting the women face compared to the fine-tuned version of SAM-ResNet. This is likely the reason explaining why SAM-ResNet model outperforms the fine-tuned one. Beyond the correlation coefficient, we also observe a similar trend in gain performance for the other tested metrics.

### Data accessibility

The list of paintings (title, artist, year, art movement and link) is given in S1 File. The year is either the year the painting has been made or the date of birth and death of the artist, when the exact year the painting has been made is not known. All the paintings can be downloaded from internet. The internet links to download the different paintings are provided.

We provide the following link http://www-percept.irisa.fr/art_paintings/ which allows readers to get all supporting information of this study:

- All eye-tracking data are released for the sake of reproducible research. This consists of the spatial coordinates of visual fixation as well as the fixation durations for each observer. We also provide human saliency maps and fixation maps.
- A Python script is provided in order to fit the downloaded paintings to the desired resolution, *i.e.* 1600 × 900. By maintaining the aspect ratio of the painting, we first added grey stripes (*RGB* = (100, 100, 100)) on the top/bottom or on the left/right side and then we resized the paintings to get the final resolution, 1600 × 900.
- The predicted saliency maps for the 8 tested models are provided. Results of the fine-tuned SAM-ResNet model are also available.
- The new weights for SAM-ResNet model are also provided as well as the source code of SAM-ResNet model to reproduce all our results.

Note that most of the paintings used in this study are in the public domain under the CC BY 4.0 licence. However, we only provide the link to download paintings to avoid copyright infringement.

## Conclusion

In this paper, we performed an eye tracking experiment on 150 paintings belonging to 5 art movements, namely Fauvism, Impressionism, Pointillism, Realism and Romanticism. We found out that the gaze deployment over these paintings is very similar to the gaze deployment on natural scenes we are used to observe. As the chosen art movements illustrate daily life, this result was not so surprising. We then evaluated the performance of existing saliency models to predict where an observer would look at. Performances are rather good for deep-based models, and rather low for handcrafted models. We went further by fine-tuning an existing deep saliency model and succeeded in improving in a significant manner the prediction performance.

This new model, specialized for paintings, would allows us to design new and automatic image-based applications, such as transformation of a painting into a video sequence; it would consist in showing sequentially the most interesting part of the painting.

In future work, we would like to study the less figurative periods. It will be worth including Cubism, Expressionism, and Abstraction periods. In the same way, we could include painting movements before Italian Renaissance, such as the Early Netherlandish painting school.

## Supporting information

S1 File Paintings used in this study (in alphabetical order). In the following Tables 7, 8 and 9, we provide information regarding the paintings used in this study. It consists of the painting tittle, its author, the year, the art period and the internet link where the painting has been downloaded.
(PDF)
