# Supplementary material for: Can we accurately predict where we look at paintings?
Source: PLoS One. 2020 Oct 9;15(10):e0239980. doi: 10.1371/journal.pone.0239980 (PMC7546463; doi:10.1371/journal.pone.0239980)
Supplement: S1 File — In the following Tables 7, 8 and 9, we provide information regarding the paintings used in this study. It consists of the painting tittle, its author, the year, the art period and the internet link where the painting has been downloaded. (PDF) [file pone.0239980.s001.pdf]

Supporting information

S1 Tables. Paintings used in this study (in alphabetical order). In the following tables 1, 2 and 3, we provide information regarding the paintings used in this study. It consists of the painting tittle, its author, the year, the art period and the internet link where the painting has been downloaded.

Table 1. Paintings used in this study (in alphabetical order).

Title	Artist	Year	Movement	Link
A Highland Lanscape	Sir Edwin Henry Landseer	1830	Romanticism	Here
A Storm off the Normandy Coast	Eugène Isabey	1850	Romanticism	Here
A Study in Curves	William Merritt Chase	1890	Realism	Here
After a Rain Country Road	Fyodor Vasilyev	1867	Realism	Here
Akt Meski	Jan Matejko	1838/93	Romanticism	Here
Alfalfa, St. Denis	Georges Seurat	1885	Pointillism	Here
Automne en France	Emily Carr	1911	Fauvism	Here
Avenue of Trees in a Small Town	Alfred Sisley	1866	Impressionism	Here
Bacchante	Joaqu'n Sorolla	1886	Impressionism	Here
Bank of a Lake	Max Pechstein	1910	Fauvism	Here
Basket of Fruits	Edouard Manet	1864	Realism	Here
Bathers	Henri-Edmond Cross	1899	Pointillism	Here
Bodegon con salmon	Francisco de Goya	1812	Romanticism	Here
Bodegones con pargos dorados	Francisco de Goya	1812	Romanticism	Here
Bowl of Fruit	Henri Fantin-Latour	1857	Realism	Here
Chestnut Avenue in La Celle Saint Cloud	Alfred Sisley	1865	Impressionism	Here
Chichester Canal	Joseph Mallord William Turner	1828	Romanticism	Here
Cows at a Pond	Gerard Bilders	1856	Realism	Here
Dead Hare	James Ward	1769/1859	Romanticism	Here
Etude de femme nue, couchée sur un Divan	Eugene Delacroix	1826	Romanticism	Here
Female Nude with a Dog (Portrait of Leotine Renaude)	Gustave Courbet	1866	Realism	Here
Femme Nue et Dormant	Gustave Courbet	1858	Realism	Here
Fleurs et Fruits	Charles Camoin	1942	Fauvism	Here
Flowers and Fruit, a Melon	Henri Fantin-Latour	1865	Realism	Here
Fruit, Knife and Napkin	Albert Marquet	1899	Pointillism	Here
Garlic Cloves and Knife on the Corner of a Table	Gustave Caillebotte	1871	Impressionism	Here
Houses at Chatou	Maurice de Vlaminck	1905	Fauvism	Here
Huisje aan een sloot	Anton Mauve	1888	Realism	Here
Kirkstall Abbey, Yorkshire	Thomas Girtin	1801	Romanticism	Here
Kneeling Nude	Edgar Degas	1888	Impressionism	Here
L'Heure Embrasée	Theo van Rysselberghe	1897	Pointillism	Here
La Danse	Henri Matisse	1909	Fauvism	Here
La Fuite des Nymphes	Henri-Edmond Cross	1906	Pointillism	Here
La Grande Jatte	Georges Seurat	1884	Pointillism	Here
Lair of the Sea Serpent	Elihu Vedder	1899	Realism	Here
Landscape at l'Estaque	Georges Braque	1907	Fauvism	Here
Landscape with Cattle at Limousin	Jules Dupre	1837	Realism	Here
Landschaft mit dem mondregenbogen	Caspar David Friedrich	1810	Romanticism	Here
Le Port d'Anvers	Georges Braque	1906	Fauvism	Here
Le Puy in the Snow	Albert Dubois-Pillet	1889	Pointillism	Here
Les Deux Amies	Marie Vorobieff (Marevna)	1930	Pointillism	Here
Les Poseuses	Georges Pierre Seurat	1886	Pointillism	Here
Los Pajaros Muertos	Francisco de Goya	1812	Romanticism	Here
Luxe, Calme et Volupté	Henri Matisse	1905	Pointillism	Here
Lying Nude	Lesser Ury	1889	Impressionism	Here
Male Model Resting	John Singer Sargent	1895	Realism	Here
Man Holding a Dagger	Luis de Madrazo y Kuntz	1850	Romanticism	Here
Morning	Charles-Francois Daubigny	1858	Realism	Here

Table 2. Paintings used in this study (in alphabetical order).

Title	Artist	Year	Movement	Link
Morning in a Pine Forest	Ivan Shishkin	1889	Realism	Here
Morning, An Overcast Day, Rouen	Camille Pissarro	1896	Impressionism	Here
Mother and Child by the Sea	Johan Christian Dahl	1830	Romanticism	Here
My House	Henri Martin	1860-1943	Pointillism	Here
Naked man reversed on the ground	Theodore Gericault	1817	Romanticism	Here
Naked Woman Lying on a Couch	Gustave Caillebotte	1873	Impressionism	Here
Nasturtiums and dahlias in a basket	Paul Gauguin	1884	Impressionism	Here
Nature Morte	Maurice de Vlaminck	1910	Fauvism	Here
Nature morte	Louis Hayet	1888	Pointillism	Here
Nature Morte à la Cafetiére Rouge	Maurice de Vlaminck	1876-1958	Fauvism	Here
Nature Morte à la Coupe de Fruits et au Verre	Jean Metzinger	1905	Pointillism	Here
Nature morte à la cruche bleue	Henri Matisse	1903	Fauvism	Here
Nature Morte au Citron	Henri Matisse	1917	Fauvism	Here
Nature Morte au Panier	André Derain	1924/27	Fauvism	Here
Nature Morte aux Oranges	Maurice de Vlaminck	1909	Fauvism	Here
Nature Morte aux Raisins sur un Plat	André Derain	1944	Fauvism	Here
Nature Morte avec Bouteilles et Pains	Francisco de Goya	1826	Romanticism	Here
Niagara Falls	Philip L. Hale	1902	Impressionism	Here
Nu Couché	Maurice de Vlaminck	1905	Fauvism	Here
Odalisque	Henri Matisse	1920/21	Fauvism	Here
Odalisque	Eugene Delacroix	1825	Romanticism	Here
Oysters	Edouard Manet	1862	Realism	Here
Parrot Tulips (II)	Henri Matisse	1905	Pointillism	Here
Paysage à la Ciotat (la Pointe du Capucin)	Achille Emile Othon Friesz	1907	Fauvism	Here
Pears and Grapes	Paul Gauguin	1875	Impressionism	Here
Plage de la Vignassa	Henri-Edmond Cross	1891	Pointillism	Here
Plate of Peaches	Henri Fantin-Latour	1862	Realism	Here
Poppy Fields near Argenteuil	Claude Monet	1875	Impressionism	Here
Pots et Fruits	Henri Matisse	1901	Fauvism	Here
Rapids on the Petite Creuse at Fresselines	Claude Monet	1889	Impressionism	Here
Reclining Nude	Frederic Bazille	1860	Impressionism	Here
Reclining Nude	Pierre-Auguste Renoir	1883	Impressionism	Here
Reclining Nude	John Singer Sargent	1862	Realism	Here
Reclining Odalisque or Woman with a Parakeet	Eugene Delacroix	1827	Romanticism	Here
Red Mullets	Claude Monet	1869	Impressionism	Here
Restaurant de la Machine à Bougival	Maurice de Vlaminck	1905	Fauvism	Here
Resting Bacchante	Joaqu'n Sorolla	1887	Impressionism	Here
Rochers sur la Côte à Agay	Armand Guillaumin	1907	Fauvism	Here
Ronde	Henri-Edmond Cross	1905	Pointillism	Here
Sailboats on the River Scheldt	Théo van Rysselberghe	1892	Pointillism	Here
Sleeping Nude Woman	Gustave Courbet	1865	Realism	Here
Snow Storm - Steam-Boat off a HarbourÕs Mouth	Joseph Mallord William Turner	1842	Romanticism	Here
Standing sitter with a stick. Sitter on a rock	Orest Kiprensky	1801	Romanticism	Here
Still Life	Camille Pissarro	1869	Impressionism	Here
Still Life	Henri Matisse	1869-1954	Pointillism	Here
Still life of Sheep's Ribs and Head - The Butcher's conter	Francisco de Goya	1812	Romanticism	Here
Still Life Post, Bottle, Cup and Fruit	Paul Cezanne	1871	Impressionism	Here
Still Life With A Purro (II)	Henri Matisse	1904	Pointillism	Here
Still Life with Bottle of Wind	Henri-Edmond Cross	1904	Pointillism	Here
Still Life with Fish	Frederic Bazille	1866	Impressionism	Here

Table 3. Paintings used in this study (in alphabetical order).

Title	Artist	Year	Movement	Link
Still Life with Jugs and Pipe	Georges Braque	1906	Fauvism	Here
Still Life with Lobsters	Eugene Delacroix	1826	Romanticism	Here
Still Life with Peaches and Grapes	Pierre-Auguste Renoir	1881	Impressionism	Here
Still Life with Pumpkin, Plums, Cherries, Figs and Jug	Theodule Ribot	1860	Realism	Here
Still life with Red Mullet and Jug	Paul Gauguin	1876	Impressionism	Here
Still Life with Skate	Eugene Boudin	1861	Realism	Here
Still Life with White Pitcher	Adolphe Joseph Thomas Monticelli	1822	Romanticism	Here
Still life, Plucked Turkey and Pan with Fish	Francisco de Goya	1812	Romanticism	Here
Still Life: Fruits on a Table	Edouard Manet	1864	Realism	Here
Still Life: Heron with Spread Wings	Alfred Sisley	1867	Realism	Here
Stilleven Met Vis	Albert Dubois-Pillet	1885	Pointillism	Here
Study of a Reclining Nude	Eugene Delacroix	1824	Romanticism	Here
The Augustan Bridge at Narni	Camille Corot	1826	Realism	Here
The Banks of the Marne at Dawn	Albert Dubois-Pillet	1888	Pointillism	Here
The Banks of the Seine at Neuilly	Albert Dubois-Pillet	1886	Pointillism	Here
The Bather	Jean-Francois Millet	1848	Realism	Here
The Bathers	Gustave Courbet	1858	Realism	Here
The Berlin-Potsdam Railway	Adolph Menzel	1847	Realism	Here
The Blue Nude (Souvenir of Biskra)	Henri Matisse	1907	Fauvism	Here
The Farm at Les Collettes, Cagnes	Auguste Renoir	1908	Impressionism	Here
The Forest	Henri-Edmond Cross	1907	Pointillism	Here
The Gardener	Maurice de Vlaminck	1904	Fauvism	Here
The Girl from Rat Mort	Maurice de Vlaminck	1905	Fauvism	Here
The Joy of Life	Henri Matisse	1906	Fauvism	Here
The Kiss	Theodore Gericault	1816	Romanticism	Here
The Large Bathers	Pierre-Auguste Renoir	1887	Impressionism	Here
The Luxembourg Gardens	Henri Matisse	1901	Fauvism	Here
The Lying Nude	Henri Matisse	1906	Fauvism	Here
The Model's Siesta	Théo van Rysselberghe	1920	Pointillism	Here
The Orchard	Maurice de Vlaminck	1905	Fauvism	Here
The Pine Tree at St. Tropez	Paul Signac	1909	Pointillism	Here
The Pointe de per Kiridec at Roscoff	Theo van Rysselberghe	1889	Pointillism	Here
The Rainbow	Henry Moore	1865	Realism	Here
The Reclining Shepherdess	Berthe Morisot	1891	Impressionism	Here
The Salmon	Edouard Manet	1868	Realism	Here
The Sleepers	Gustave Courbet	1866	Realism	Here
The Tea Set	Claude Monet	1872	Impressionism	Here
The Three Skulls	Theodore Gericault	1814	Romanticism	Here
The Two Models	Raoul Dufy	1930	Fauvism	Here
The Wood	Henri-Edmond Cross	1905	Pointillism	Here
Trouville, Black Rocks	Eugene Boudin	1863	Impressionism	Here
Two sitters	Orest Kiprensky	1800	Romanticism	Here
Un Jardin, Maison Baptiste	Theodore Earl Butler	1895	Impressionism	Here
Upper mississipi	John Frederick Kensett	1855	Impressionism	Here
Vase of Flowers	Theo van Rysselberghe	1907	Pointillism	Here
Vase of Flowers in a Window	Henri Martin	1860-1943	Pointillism	Here
Venice, from the Porch of Madonna della Salute	Joseph Mallord William Turner	1835	Romanticism	Here
Vesuvius from Posillipo by Moonlight	Joseph Wright of Derby	1774	Romanticism	Here
Waves Breaking against the Wind	Joseph Mallord William Turner	1840	Romanticism	Here
Woman at Her Toilette	Edgar Degas	1876	Impressionism	Here
Woman with a Parrot	Gustave Courbet	1866	Realism	Here
Young Girls Playing with a Lion Cub	Louis Valtat	1905	Fauvism	Here
